# Mental health and quality of life outcomes in family members of patients with chronic critical illness admitted to the intensive care units of two Brazilian hospitals serving the extremes of the socioeconomic spectrum

**DOI:** 10.1371/journal.pone.0221218

**Published:** 2019-09-13

**Authors:** Renata Rego Lins Fumis, Antonio Bento Ferraz, Isac de Castro, Henrique Souza Barros de Oliveira, Marcelo Moock, José Mauro Vieira Junior

**Affiliations:** 1 Intensive Care Unit, Hospital Sírio-Libanês, São Paulo, Brazil; 2 Intensive Care Unit, Hospital Geral do Grajaú, São Paulo, Brazil; 3 Division of Nephrology and Molecular Medicine, Department of Medicine, University of São Paulo School of Medicine, São Paulo, Brazil; Hospital Israelita Albert Einstein, BRAZIL

## Abstract

Chronic critical illness (CCI) is a relevant clinical, social and financial health issue. The aim of this study was to compare the mental outcomes (symptoms of anxiety and depression) and quality of life outcomes of the family members of patients with CCI from different socioeconomic backgrounds who were admitted to one of the intensive care units (ICUs) in two Brazilian hospitals, one private and one public. It is a prospective study involving a public hospital that serves a low-income population and a tertiary private hospital that serves a high-income population. Family members of patients with CCI answered the Hospital Anxiety and Depression Scale (HADS) and The World Health Organization Quality of Life–WHOQOL-bref questionnaires. They responded to the European Quality of life Five Dimension three Level (EuroQol-5D-3L) and the Activities of Daily Living (ADL) questionnaires on behalf of the patients at three time points: during the ICU stay, 30 and 90 days after the patient was discharged. We used logistic regression models to evaluate the main predictors of a binary outcome regarding symptoms of anxiety and depression. We enrolled 186 patients with CCI. Many patients from public hospitals who were independent became dependent for their ADLs at 90 days (41.7% versus 14.3%, p = 0.03). At 30 days, family members from public hospital had worse impact on all domains of WHOQOL-bref compared with families from private hospital. At 90-days, the difference persists in the physical domain, worse for families from public hospital (p = 0.006). The symptoms of depression at 30-days (p = 0.008) and at 90-days (p = 0.013) were worse in the public hospital. CCIs affected quality of life and the emotional condition of family members, especially in families with fewer resources when the patients became more dependent. Family members with higher education were more likely to experience depression, while depression was associated with cohabiting with the patient in low-income families.

## Background

Chronic critical illness (CCI) impacts a growing population of patients who have survived acute critical illnesses. [1–4]. In 2015, the ProVent study introduced a new definition of CCIs, that was patients who have spent at least 8 days in an ICU and who have presented with one of the six eligible clinical conditions: mechanical ventilation for at least 96 hours in a single episode; tracheotomy; sepsis or other severe infections; extensive wounds; stroke; and traumatic brain injury. This definition contrasts with the formerly used definition of CCI, which required a longer period of mechanical ventilation of up to 21 days [2].

The patient’s critical illness is a stressful event that modifies the family structure, and the prolonged state and severity of the illness add to the instability of the family structure. CCI has been acknowledged as a significant clinical, social and financial health issue, and it indeed provokes major changes in the patients’ QOL and the family environment [2–4]. Symptoms of psychological distress affect more than half of family members impacted by the patient’s CCI.

Among the family members of patients requiring at least 48 hours of mechanical ventilation, one-third (34%) were at risk of clinical depression, due the perception of the patient’s functional status after hospital discharge, among other problems [5, 6].

Unfortunately, most of the information regarding the impact of CCI comes from developed countries, and there is a lack of information on the social and economic burden in less developed countries [7].

Due to high hospitalization costs and poor long-term survival, the clinical and financial burden of CCI is expected to increase in the coming years because of an aging population and advances in the early management of critical illness, leading to more long-term survivors [2]. Therefore, it is crucial to develop strategies to prevent CCIs, reduce their negative effects, and reduce costs [8], especially in less developed countries in which socioeconomic deprivation is more common and 80% of all deaths are due to chronic disease [9]. In Brazil, a better understanding of CCI may help the official hospital system improve the care provided to patients and their family members with low socioeconomic status.

The purpose of this study was to compare the mental outcomes (symptoms of anxiety and depression); to identify predictors of mental health outcomes and QOL outcomes in family members of patients with CCIs from different socioeconomic backgrounds who were admitted to the intensive care unit (ICU) in one of two Brazilian hospitals, one private and one public, at the extremes of the social pyramid.

A public hospital in Brazil serves a primarily low-income population, which represents the largest number of patients assisted by the Public Hospital System–SUS. Only approximately thirty percent of Brazilians have private health insurance, and these individuals with private insurance are concentrated in the urban areas of the southeastern part of the country [10]. The two hospitals have the same administrator and consequently have the same admission policy. However, these hospitals serve distinct populations with completely different socioeconomic resources. According to the City Hall of São Paulo, there are severe inequalities between the districts of “Bela Vista” and “Grajaú”, such as the number of jobs per thousand inhabitants, which is much lower in the “Grajaú” neighborhood (19,131.51 versus 471.28). The “Grajaú” neighborhood is considered poorer compared to “Bela Vista”, with the worst public policy indicators, for example, given the percentage of houses in shanty town over the total number of houses in the region (16.77 versus 0.0). [11]. The ranking of Human Development Index (HDI) in “Grajaú” district is lower (0.6) than HDI of districts of “Bela Vista (HDI = 0.8) where it is located the private Hospital “Sírio-Libanês. Furthermore, the demographic characteristics of the patients from the public hospital are characterized by a median age of 59 years, 50% of men, while the private hospital serves older patients, with a median age of 69 years and 68% of males.

## Methods

This prospective study was conducted in two hospitals attending patients of different socioeconomic levels: a public hospital, “Hospital Geral do Grajaú”, with a 25-bed ICU located in a southern suburb and serving a population with low socioeconomic status, and a tertiary private hospital, “Hospital Sírio-Libanês”, with a 30-bed ICU located in Bela Vista, the central region, and attending a population with high socioeconomic status. Both hospitals are located in the city of São Paulo.

The Institutional Review Board (IRB), called “Comitê de Ética em Pesquisa da Sociedade Beneficiente de Senhoras do Hospital Sírio-Libanês”, reviewed and approved this study (HSL protocol number HSL 2015–27, 04/16/2015).

### Participants of the study

To assess CCI status, we used the definition proposed by the investigators from the ProVent study: patients who spent at least 8 days in an ICU and who presented with one of the six eligible clinical conditions: mechanical ventilation for at least 96 hours in a single episode, tracheotomy, sepsis or other severe infections, extensive wounds, stroke, and traumatic brain injury. The exclusion criteria were patients with moribund status with a life expectancy ≤ 48 hours; patients admitted to the ICU after head and neck surgery with protective tracheotomy; and patients who were readmitted. For each patient, one family member (spouse, offspring, parent, brother) who was identified as the family member most likely to be involved with the patient’s care was included in this study. The exclusion criteria for family members were psychiatric disorders and drug treatment for anxiety and depression.

Family members answered the Hospital Anxiety and Depression Scale (HADS) and the WHOQOL-BREF while in the ICU. At 30 and 90 days after ICU discharge, they were also interviewed by phone to complete the WHOQOL-BREF and HADS. Only those who completed the questionnaires in the ICU were included in the follow-up. The HADS score for each subscale (anxiety and depression) ranged from 0 to 21, and a cut-off of > 10 was used to identify each condition. [12] Scores for the entire scale range from 0–42, with higher scores indicating more distress. The WHOQOL-BREF contains a total of 26 questions. The four domain scores denote an individual’s perception of quality of life in each particular domain. Domain scores (Physical health, Psychological, Social relationships, and Environmental) are scaled in a positive direction, 0–130 points (higher scores denote higher quality of life).

The EQ-5D-3L is calculated from patient scoring of five dimensions (mobility, self-care, usual activities, pain/discomfort, anxiety/depression). For each dimension, participants are asked to mark between 1: ‘no problems’ to 3: ‘unable to/extreme problems’. Total score range from 5 to 15 points. Katz Index of Independence in Activities of Daily Living: 0–6 points possible; 6 points = independent; 3–5 = partially dependent; 2 or less points = dependent.

At the ICU and 30 and 90 days after first interview done at ICU, the family member responded to the EQ-5D-3L and the ADL for the patient to assess the patient’s independence level (6 = high patient independence to 0 = patient is very dependent). All scales were previously validated in Brazil [13–16] and validated for completion by proxies [17,18]

### Data collection

For each patient, the following information was collected: age, gender, marital status, level of education, cause of ICU admission, comorbidities, dementia, delirium, Simplified Acute Physiology Score 3 (SAPS 3), Glasgow Coma Scale score, Sequential Organ Failure Assessment (SOFA) score, length of stay (LOS), mechanical ventilation requirement, vasopressors, renal replacement therapy (RRT), blood transfusion, delirium (positive CAM-ICU), palliative care, and final outcome. The following information was supplied by the family member: gender, age, marital status, level of education, religious belief, monthly family income, work, relationship with the patient and previous ICU experience. Patient baseline characteristics were collected from the medical record. To ensure the optimal quality of the data, the same investigator (RRLF), a psychologist with ICU interview experience, collected all the study data and conducted all the interviews with the families.

### Statistical analysis

Normality was assessed using the Kolmogorov-Smirnov test. Continuous data are presented as the median and interquartile range (25%-75%). Categorical variables are shown as absolute values and percentages. To compare two groups using a nonparametric test, the Mann-Whitney test with the Bonferroni correction was performed. The Friedman test with the Simes-Hochberg correction was performed to compare the results from the WHOQOL-Bref, EQ-5D-3 L and HADS at the three time points. The categorical analyzes were performed through the chi-square test with the Yates correction for unbalanced samples. In the comparison between medians the tests of analysis of stations of Mann Witney was corrected by Bonferroni.

We used logistic regression models to evaluate the main predictors of a binary outcome regarding symptoms of anxiety and depression (HADS subscale). The logistic regression was performed with a sample size of 100 patients from the private hospital and 86 patients from the public hospital and included calculations of corresponding crude and adjusted odds ratios (ORs) and 95% confidence intervals (CI). The variables that were included were those that presented significance with p-values < 0.10 based on the Pearson chi-square test or the Mann-Whitney test. Once the candidate variables were inserted, the logistic regression was performed through the step-by-step model, considering that the variable with the worst influence was the one with the lowest Wald index. Numerous steps were performed until the model became cohesive and all variables were significant (p<0.05) and reached p > 0.20 in the Hosmer-Lemeshow test for model validation. Statistical analyses were carried out using SPSS for Windows version 19.0 (IBM Corp., Armonk, NY, USA). A two-sided p-value ≤ 0.05 was considered statistically significant. Canonical Analysis was performed to verify the influence of missing data on the study variables.

## Results

**Fig 1:**
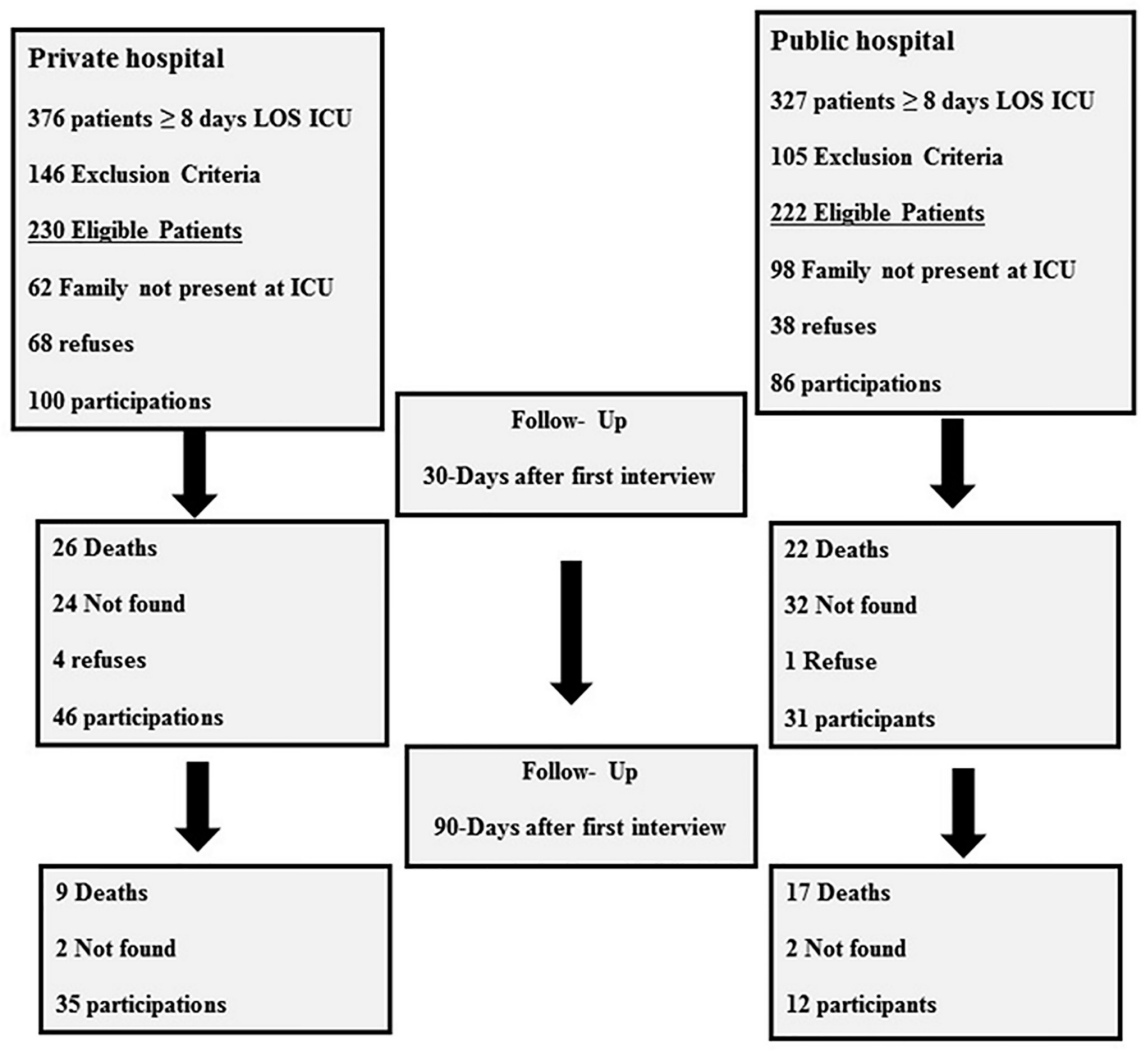
Flow chart. LOS ICU = length of stay at Intensive Care Unit.

Between May 2015 and March 2017, 100 patients from the private hospital and 86 patients from the public hospital, that met the CCI criteria, participated in this study after signing the written informed consent. The reasons for non-inclusion are outlined in the flowchart (Fig 1). The raw data of the patients of this study is available as S1 Table.

### Chronic critical illness criteria eligibility

Sepsis and mechanical invasive ventilation for four consecutive days plus at least 8 days in an ICU were the most prevalent CCI eligibility criteria in both hospitals (more than 70% for both). Patients of the public hospital underwent tracheotomy more frequently than those of the private hospital (41.9% versus 16.0%, p <0.0001).

### Patient characteristics

Patients from the public hospital were younger (58.5 [47.0–67.0] versus 69 [59.5–81] years of age; p<0.001). We observed a similar median SAPS 3 (55 [47–62] versus 54[46–64]; p = 0.689) and Charlson index (1.0 [0–3] versus 2.0[1–4]; p = 0.059) between the patients at the two institutions. A significant difference was found between the public and private hospitals in the ICU median LOS (21[14–30] versus 16 [10–26] days, respectively; p = 0.006) and in the median number of days on mechanical ventilation (13 [8–20] versus 9.0 [6–15] days, respectively; p = 0.026). Although no difference was found in EQ-5D-3L at baseline, ICU mortality was higher in the public hospital (32.6% versus 17.2%, p = 0.026), whereas there was no difference in the overall hospital mortality rates (38.1% versus 35.1%, p = 0.837) (Table 1).

**Table 1:** Clinical variables and hospital outcomes of CCI patients admitted to an ICU according to hospital, baseline.

Variables	Private hospital (N = 100)	Public hospital (N = 86)	p
Medical condition, n(%)	71 (71.0%)	73 (84.9%)	0.024
SAPS 3, median[IQR]	53.5 [45.5–63.5]	54.5 [47.0–62.0]	0.689
SOFA, median [IQR]	6.0 [3.0–9.0]	4.0 [2.0–8.0]	0.062
Charlson, median [IQR]	2.0 [0.5–4.0]	1.0 [0.0–3.0]	0.059
Glasgow, median [IQR]	14.0 [13.0–15.0]	15.0 [13.0–15.0]	0.337
ADL, median [IQR]	6 [2.0–6.0]	6 [4.7–6.0]	0.023
WHOQOL-BREF Environment	87.5 [75.8–93.8]	62.5 [46.9–78.1]	<0.0001
WHOQOL-BREF Physical health	78.6 [67.9–92.9]	71.4 [58.9–85.7]	0.015
WHOQOL-BREF Psychological	75.0 [62.5–87.5]	70.8 [58.3–83.3]	0.042
WHOQOL-BREF Social	83.3 [66.7–100]	75.0 [58.3–83.3]	0.001
HADS total score, baseline	15.0 [9.3–21]	19.5 [14–26]	0.003
HADS A score, baseline	9.0 [7.0–13.0]	11.0 [8.0–14.0]	0.008
HADS D score, baseline	6.0 [3.0–9.0]	8.0 [5.0–11.0]	0.003
EQ-5D-3L, median [IQR]	7.0 [6.0–10.0]	7.0 [6.0–10.0]	0.651
Dependence ADL, n(%)	24 (24.0%)	10 (11.6%)	0.030
Blood transfusion, n(%)	47 (47.0%)	32 (37.2%)	0.178
RRT, n(%)	22 (22.0%)	26 (30.2%)	0.201
Delirium, n(%)	54 (54.0%)	10 (11.6%)	<0.0001
Vasopressor, n(%)	86 (86.0%)	63 (73.3%)	0.030
Cancer, n(%)	45 (45.0%)	4 (4.7%)	<0.0001
Palliative Care, n(%)	9 (9.0%)	9 (10.5%)	0.736
Demency, n(%)	11 (11.0%)	2 (2.3%)	0.021
ICU LOS, median [IQR]	16.0 [10.0–26.0]	20.5 [14.0–30.0]	0.006
ICU death, n(%)	17 (17.2%)	28 (32.6%)	0.026
Hospital LOS, median [IQR]	38.0 [27.0–56.0]	35.0 [23.0–54.0]	0.309
Hospital death, n (%)	34 (35.1%)	32 (38.1%)	0.837
Hospital readmission, n(%)	50 (50.0%)	24 (27.9%)	0.002
ICU readmission, n(%)	32 (32.0%)	10 (11.6%)	0.001

SAPS, Simplified Acute Physiology Score; SOFA, Sequential Organ Failure Assessment; ADL, Activities of Daily Living; EQ-5D-3L = European Quality of Life Five Dimension Three Level; RRT, Renal Replacement Therapy; ICU, Intensive Care Unit; LOS, Length of Stay; WHOQOL-BREF: World Health Organization Quality of Life; HADS, Hospital Anxiety and Depression Scale; HADS A- Anxiety subscale; HADS D, Depression subscale; IQR, interquartile range.

### Family member characteristics

In the public hospital, 91.8% of families had a monthly income less than $1,000, while most families of the patients in the private hospital (88%) had a monthly income greater than $2,400, p<0.001; in the public hospital, 32.6% of the family members of the patients were unemployed versus 1.0% in the private hospital (p<0.001) (Table 2).

**Table 2:** Patient´s and family member’s demographic characteristics according to hospital.

Demographic variables	Private hospital N = 100	Public Hospital N = 86	p
**Patients**			
**Age, median [IQR]**	69 [59.5–81.0]	58.5 [47.0–67.0]	<0.0001
**Gender, n(%)**			0.164
Male	68 (68.0%)	50 (58.1%)	
**Marital status, n(%)**			0.016
Single	73 (73.0%)	48 (55.8%)	
Married	11 (11.0%)	21 (24.4%)	
**Level of education, n(%)**			<0.001
Elementary school	12 (12.0%)	57 (66.3%)	
High school	21 (21.0%)	26 (30.2%)	
College education	67 (67.0%)	3 (3.5%)	
**Religion, n(%)**			<0.001
Catholic	71 (71.0%)	45 (52.3%)	
Protestant	2 (2.0%)	29 (33.7%)	
Others	27 (27.0%)	12 (14.0%)	
**Family members**			
**Age, median [IQR]**	58.0 [47.5–67.0]	38.0 [31.0–52.0]	<0.0001
**Gender Male, n(%)**	17 (17.0%)	29 (33.7%)	0.008
**Level of education, n(%)**			<0.0001
Elementary school	4 (4.0%)	33 (38.4%)	
High school	21 (21.0%)	39 (45.3%)	
College Education	81 (81.0%)	14 (16.3%)	
**Monthly family income, n(%)**			
Less than $1,000	4.0 (4.0%)	79 (91.8%)	<0.001
**Work, n(%)**			
Unemployed	1.0 (1.0%)	28 (32.6%)	<0.001
**Marital status, n(%)**			0.001
Single	11(11.0%)	24 (27.9)	
Married	82 (82.0%)	51 (59.3)	
**Relationship, n(%)**			<0.0001
Offspring	36 (36.0%)	44 (51.2%)	
Spouses	47 (47.0%)	19 (22.1%)	
**Previous ICU experience, n(%)**	75 (75.0)	39 (45.3)	<0.0001

ICU, Intensive Care Unit; IQR, Interquartile Range.

### Quality of life of family members

**Fig 2:**
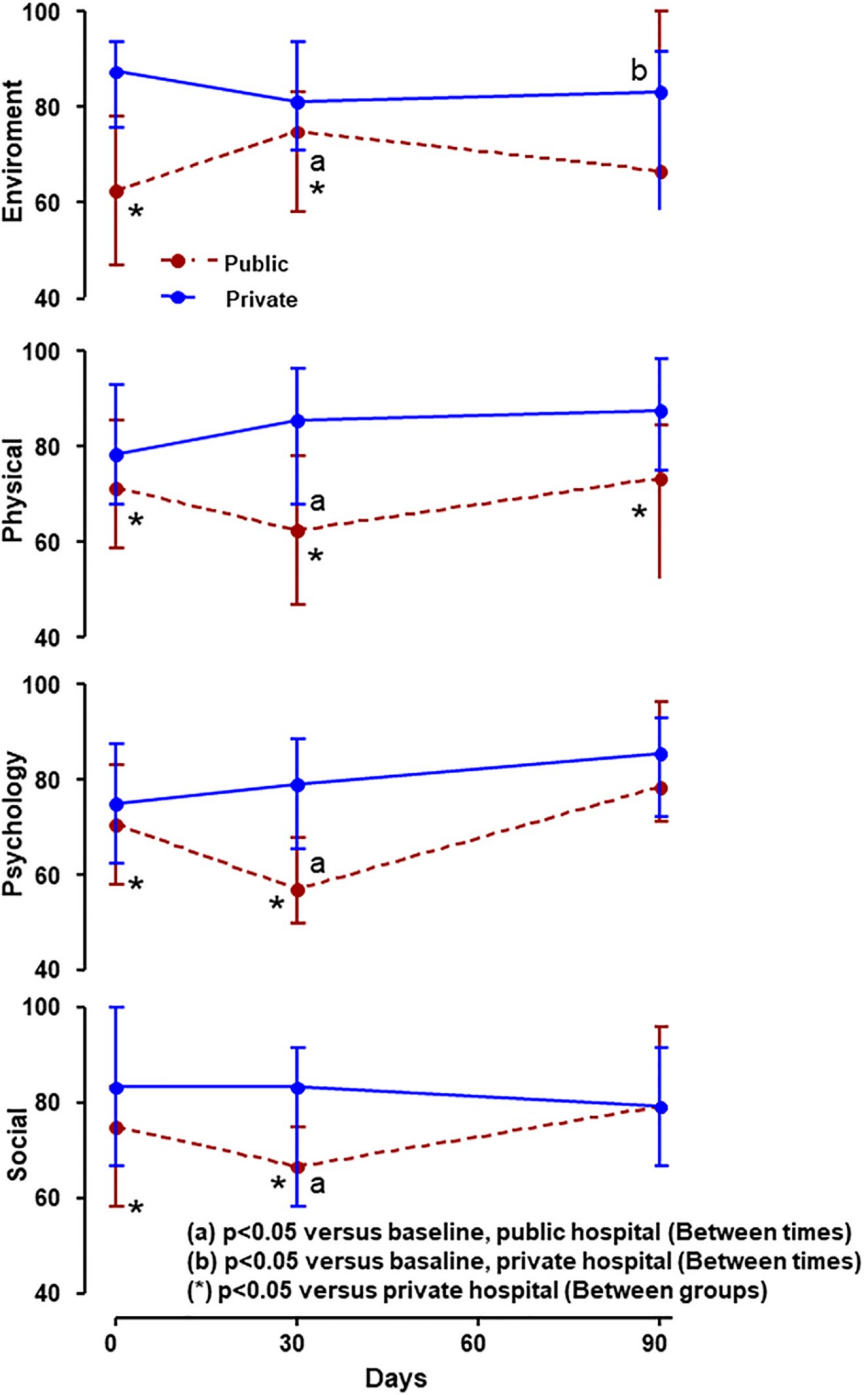
WHOQOL-Bref at three time points according to the hospital in which the patient was treated. Data were expressed in medians and quartiles. The comparison between the times used the Friedman test with post-SNK test. In the comparison between the groups, the Mann-Whitney test.

Family members of patients in the private hospital showed significant difference in all whoqol-bref domains at baseline compared to family members from public hospital. Over time, the only difference observed was in the environmental domain with a significant decrease at 90 days compared to their baseline values (p = 0.043). In the public hospital, families showed a decrease in all domains at 30-days after first interview at ICU: environment (p<0.0001); physical (p<0.0001), psychological (p<0.0001) and social (p = 0.004). However, these domains returned to their baseline values at 90 days after first interview at ICU. It was observed that the physical domain was the only one that remained significantly worse when compared to the families of patients treated at private hospital (p = 0.006), (Fig 2 and Table 3).

**Table 3:** Analysis of HADS, WHOQOL-BREF, EQ-5D-3L and ADL over time (Baseline, 30 and 90 days) at the private and public hospital.

Outcomes	Private hospital	P ^a^	Public hospital	P ^a^	p ^b^
**HADS Total Score**					
HADS total score, baseline	15.0 [9.3–21]		19.5 [14–26]		0.003
HADS total score, 30-days	10.0 [2.8–15.3]	<0.0001	11.0 [3–23]	<0.0001	0.451
HADS total score, 90-days	5.0 [1.0–12.0]	<0.0001	9.5 [1–25]	<0.0001	0.548
**HADS–A score**					
HADS A score, baseline	9.0 [7.0–13.0]		11.0 [8.0–14.0]		0.008
HADS–A score, 30 days	5 [2.0–8.0]	<0.0001	7.0 [3.0–10.0]	0.001	0.539
HADS-A score, 90 days	3.0 [1.0–6.0]	<0.0001	5.0 [1.0–12.0]	0.020	0.461
**HADS- D score**					
HADS D score, baseline	6.0 [3.0–9.0]		8.0 [5.0–11.0]		0.003
HADS-D score, 30-days	4.0 [0.0–7.0]	<0.0001	4.0 [0.0–13.0]	<0.0001	0.497
HADS-D score, 90-days	2.0 [0.0–6.0]	<0.0001	5.0 [0.0–13.0]	0.004	0.215
**WHOQOL-BREF**					
**Environment**					
Environment, baseline	87.5 [75.8–93.8]		62.5 [46.9–78.1]		<0.0001
Environment, 30-days	81.3 [71.1–93.8]	0.664	75 [58.3–83.3]	<0.0001	0.002
Environment, 90-days	83.3 [58.3–91.7]	0.043	66.7 [58.3–100]	0.057	0.891
**Physical health**					
Physical health, baseline	78.6 [67.9–92.9]		71.4 [58.9–85.7]		0.015
Physical health, 30-days	85.7 [67.9–96.6]	0.632	62.5 [46.9–78.1]	<0.0001	<0.0001
Physical health, 90-days	87.5 [75.0–98.4]	0.098	73.4 [52.3–84.4]	0.405	0.006
**Psychological**					
Psychological, baseline	75.0 [62.5–87.5]		70.8 [58.3–83.3]		0.042
Psychological, 30-days	79.2 [65.6–88.5]	0.761	57.1 [50.0–67.9]	<0.0001	0.000
Psychological, 90-days	85.7 [72.3–92.9]	0.217	78.6 [71.4–96.4]	0.195	0.510
**Social**					
Social, baseline	83.3 [66.7–100]		75.0 [58.3–83.3]		0.001
Social, 30-days	83.3 [58.3–91.7]	0.532	66.7 [58.3–75.0]	0.004	0.001
Social, 90-days	79.2 [66.7–91.7]	0.864	79.2 [66.7–95.8]	0.159	0.866
**EQ-5D-3L**					
EQ-5D-3L, baseline	7.0 [6.0–10.0]		7.0 [6.0–10.0]		0.651
EQ-5D-3L, 30-days	11.0 [8.8–12.0]	<0.0001	11.0 [7.0–13.0]	0.034	0.992
EQ-5D-3L, 90-days	9.0 [7.0–11.0]	0.030	10 [6.0–13.0]	0.553	0.507
**ADL**					
Indice de Katz—basal	6.0 [2.0–6.0]		6.0 [4,75–6,00]		0.023
Indice de Katz–30D	2.0[0.0–5.0]	<0.001	2.0 [0.0–6.0]	<0.001	0.509
Indice de Katz -90D	4.0[1.0–6.0]	0.061	1.0[0.0–6.0]	0.209	0.306
**ADL dependence, n(%)**					
Baseline	24 (24%)		10 (11.6%)		0.029
30 days	22 (47.8%)		14(45.2%)		0.818
90 days	10 (28.6%)		7(58.3%)		0.064

Median [25 percentiles -75 percentiles], Friedmann test, p<0.005 with Simes-Hochberg post-test.

^a^ Difference 30-days and 90-days versus baseline;

^b^ Difference between hospitals. HADS-A = HADS Anxiety subscale score; HADS-D = HADS Depression subscale score; WHOQOL-BREF = World Health Organization Quality of Life, EQ-5D-3L = European Quality of Life Three Dimension Five Level and ADL = Activities of Daily Living; Private

### Emotional disorders of family members

**Fig 3:**
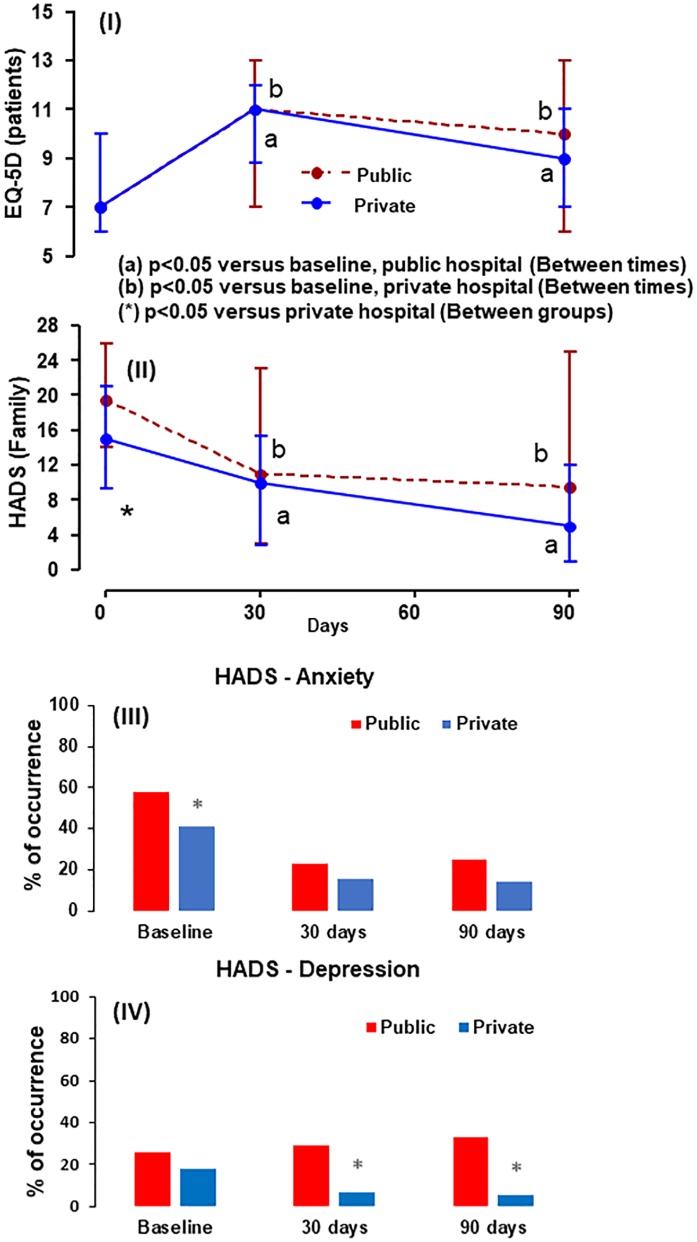
EQ-5D-3L and HADS at three time points according to the hospital in which the patient was treated. Data were expressed in medians and quartiles. The comparison between the times used the Friedman test with post-SNK test. In the comparison between the groups, the Mann-Whitney test.

At baseline, the family members of patients treated in the public hospital had a higher total HADS score (p = 0.003); Higher anxiety score (p = 0.008) and depression score (p = 0.003) compared with the family members of the patients treated in the private hospital. We observed that these scores had a significant improvement within each group over time, although family members of the public hospital presented more symptoms of depression over time: (29% versus 6.5%, p = 0.008) at 30-days and (33.3% versus 5.7%, p = 0.013) at 90-days, public versus private hospital respectively. (Fig 3 and Table 3).

### EQ-5D-3L

Concerning the EQ-5D-3L, we observed similar results between the public and private hospitals, and patients from both hospitals presented worse EQ-5D-3L scores 30 and 90 days after discharge compared to their baseline scores (Fig 3).

### ADL

Regarding ADL, we observed significant difference at baseline, showing that patients from private hospital were more dependent than those from public hospital (24% versus 11.6%, p = 0.029). We also observed that 41.7% of public hospital patients became dependent at 90-days versus 14.3% of the private hospital patients (p = 0.031).

The logistic regression revealed that while in the public hospital, the main factor associated with anxiety was the lack of previous ICU experience (p = 0.012, OR, 2.45 CI 1.22–4.95); in the private hospital, anxiety was associated with atheism (p <0.0001, OR, 2.1; CI 1.1–3.97). Regarding symptoms of depression, in the private hospital, the family members with higher education levels experienced greater levels of depression (p<0.0001, OR, 8.4; CI 2.7–26.5), and in the public hospital, cohabiting with the patient was the main factor that was strongly associated with depression (p = 0.001, OR, 8.82; CI 2.48–31.39).

## Discussion

This study sheds light on the important topic of CCI in patients and its impact on family members with distinct socioeconomic status from extremes of the social pyramid. Very little data on CCI has been collected in low-resource settings. Notwithstanding the similar comorbidities and severity, the impact of CCI in patients on the emotional state and QOL of family members varies in accordance with the resources of the family.

There were significant differences in the demographic characteristics between the two hospitals: the family members of the patients treated at the public hospital had low income, were unemployed, and experienced greater emotional suffering and poorer QOL. In accordance with the literature, the burdens of CCI are overwhelming and require considerable resources [19]; given these circumstances, families with low income may not be able to support their family members with CCI. Furthermore, increases in family demands seem to be an important indicator of the amount of assistance a family may need [20]. Interestingly, it was observed that emotional suffering was proportional to the scarcity of resources; that is, families with low income were more prone to suffer from emotional problems and experience worse QOL. In the present study, we noted this impact on low-income family members, especially those who cohabited with the patient. According to a previous study, cohabiting with the patient may increase demands on the family and decrease the strengths and capabilities (hardiness, resources, coping, and problem-solving communication) associated with outcomes of family well-being and adaptation [20].

After ICU discharge, chronic critical patients continue to require a high and continuous level of care, especially those who have become functionally dependent. This prolonged burden of care brings a severe emotional stressor to the whole family system. As a consequence, Post-Intensive Care Family Syndrome may occur in family members, including depression, anxiety, and post-traumatic stress disorder.[3,21,22] For their families, the prolonged state of critical illness triggers a number of concerns, such as role shifts, financial difficulty and disruptions in the daily activities or work schedules, and instability on family structure. [21,22] Our data show that it seems to be worse for those who cohabit with patients and are low-income financially.

CCI is a serious problem that is increasing in the health care system. The definition of this complex syndrome varies greatly, reflecting a lack of consensus. Most studies require tracheotomy or mechanical ventilation for more than 21 days [19], but we elected to use a more recent definition. [2] As such, sepsis and prolonged mechanical ventilation for at least four days were the most common CCI eligibility criteria. The experiences of family members of patients with sepsis are stressful and cause anxiety, depression and posttraumatic stress due to their helplessness and uncertainty. In addition, the death of patients or patients’ survival with significant deterioration in their QOL causes a major psychological impact on the family [23].

In our study, the ICU mortality rate was much higher than the common rate of approximately 10%, but the ICU mortality rate in our study is in accordance with the results of other CCI studies showing that, in this group of patients, one-year mortality rates range between 50 and 70%. Some studies report that only 10% of CCI patients achieve functional autonomy and live at home one year after the onset of the acute condition requiring ICU admission [1]. Furthermore, the SAPS 3 was higher than that in other Brazilian ICUs. In a retrospective cohort study in which 48,816 adult patients were admitted to 72 Brazilian ICUs, the mean SAPS 3 was 44.3 ± 15.4 points, and the ICU and hospital mortality rates were 11.0% and 16.5%, respectively [24].

Families experience uncertainties because of changes in the state of CCI patients, demands for resources and a sudden drop in QOL [3, 25]. Similarly, these family members experience mental complications (major depression, complicated mourning, acute stress disorder and posttraumatic stress disorder), which is referred to as post intensive family syndrome (PICS-F). However, PICS-F must be further explored in this specific population [26].

In the public hospital, less than half of the family members had previous knowledge of an ICU, demonstrating the scarce access to the hospital network of low-income families in Brazil. The low-income patients had fewer hospital readmissions than the high-income patients. Interestingly, among the low-income families, cohabiting with the patient was strongly associated with symptoms of depression. Health literacy, a term introduced in the 1970s that is of increasing importance in public health, is concerned with the capacities of people to meet the complex demands of health in a modern society [27]. The cognitive and social skills that determine the motivation and ability of individuals to gain access to understand and use information in ways that promote and maintain good health may be compromised in low-income families. In view of the eventual changes in lifestyle, it is important to support these families. For example, a patient with tracheotomy impacts the QOL of the family, and tracheotomy was, precisely, the most common intervention in the patients in the public hospital [28].

There are some limitations to this study. It was performed in only two hospitals, and a relatively small number of individuals were enrolled. The main limitation is that half of the families did not respond to the follow-up 30 days after ICU discharge, regardless of which hospital treated the patient. While there was no significant difference in sociodemographic factors between the nonresponders and responders, the LOS at the hospital was significantly longer in the responders than in the nonresponders (43 days [30–67] versus 33 days [25–47], p = 0.015). Given that the distribution of the missing data occurred in an aleatory way, therefore not influencing the results of the analysis at the follow up regarding the demographic variables and on the psychometric instruments applied. Nevertheless, the high number of participants lost to follow-up was due to difficulties in contacting the participants and the death of the patient; therefore, these conclusions must be viewed with caution. An important limitation is that a pre-ICU quality of life and mental health measure was not included. Therefore, we cannot say clearly whether the study findings are actually capturing the preexisting precarious health trajectory of family members from lower socioeconomic groups.

Finally, due to the severe condition of patients with CCI, only their family members were approached to fill out the questionnaires. Perhaps their perception of patient QOL was different from their own perceptions; thus, the real impact of CCI on the QOL of patients was not explored in this study.

We definitely agree that we might have captured the social gradient concept. In our results, it was observed that a third of the members of the low-income family were unemployed. Therefore, although quality of life and mental health of family members before the ICU were not addressed, our results are in agreement with previous studies [22,29] who suggested that there are inequalities in psychological suffering due to employment status, less social support and levels of financial deprivation, and thus, those with low income, low education or no job are at greater risk of depression. Our results confirm previous assumptions and findings that point to a relationship between socioeconomic status and health [22,29]. This study adds in the literature information regarding the impact of the economic and social status on the quality of life and on the emotional of the family members of the critically ill patients after ICU.

Considering the unfavorable outcomes for the majority of CCI patients and the impact on the lives of their family members, other CCI dimensions should be better evaluated, such as further research on topics as muscle, immune, and cognitive dysfunctions in order to test whether targeted therapies could conceivably improve long-term outcome, regarding QOL. Also, future research should now focus on potential initiatives tackling the CCI early on, such as applying daily diaries, cognitive behavior therapy, and mindfulness training, for instance.

## Conclusions

This study showed that the impact of CCI on the emotional status and the QOL of the family members of the patients with CCI differ according to socioeconomic resources. Cohabiting with the patient was a factor associated with family depression, while in the high-income population we found that the high level of education impacts the emotional response of the family members. Moreover, in the public hospital, the main factor associated with anxiety was the lack of previous ICU experience.

## Supporting information

S1 Table This is the data availability of the patients included in this study. (XLSX)

## Abbreviations

ADL: Activities of Daily Living
CAM-ICU: Confusion Assessment Method for the Intensive Care Unit
CCI: Chronic critical illness
CI: confidence intervals
EQ-5D-3L: EuroQol five dimensions five levels
HADS: Hospital Anxiety and Depression Scale
HSL: Hospital Sírio-Libanês
ICU: Intensive care unit
IRB: Institutional Review Board
LOS: length of stay
ORs: odds ratios
PICS-F: post intensive family syndrome
QOL: Quality of life
RRT: renal replacement therapy
SAPS 3: Simplified Acute Physiology Score 3
SOFA: Sequential Organ Failure Assessment
SUS: “Sistema Único de Saúde” e.g., Official Hospital System
WHOQOL-BREF: The World Health Organization Quality of Life
