# Correction: Mental health and quality of life outcomes in family members of patients with chronic critical illness admitted to the intensive care units of two Brazilian hospitals serving the extremes of the socioeconomic spectrum

**DOI:** 10.1371/journal.pone.0225235

**Published:** 2019-11-14

**Authors:** Renata Rego Lins Fumis, Antonio Bento Ferraz, Isac de Castro, Henrique Souza Barros de Oliveira, Marcelo Moock, M. V. Vieira Junior

The sixth author’s name is listed incorrectly in the citation. The correct citation is: Fumis RRL, Ferraz AB, de Castro I, Barros de Oliveira HS, Moock M, Vieira Junior MV (2019) Mental health and quality of life outcomes in family members of patients with chronic critical illness admitted to the intensive care units of two Brazilian hospitals serving the extremes of the socioeconomic spectrum. PLoS ONE 14(9): e0221218. https://doi.org/10.1371/journal.pone.0221218
